# Association between Severe Upper Limb Spasticity and Brain Lesion Location in Stroke Patients

**DOI:** 10.1155/2014/162754

**Published:** 2014-05-25

**Authors:** Alessandro Picelli, Stefano Tamburin, Francesca Gajofatto, Giampietro Zanette, Marialuigia Praitano, Leopold Saltuari, Claudio Corradini, Nicola Smania

**Affiliations:** ^1^Neuromotor and Cognitive Rehabilitation Research Center, Department of Neurological and Movement Sciences, University of Verona, P.le L.A. Scuro 10, 37134 Verona, Italy; ^2^Neurology Section, Department of Neurological and Movement Sciences, University of Verona, Verona, Italy; ^3^Neurology Unit, Pederzoli Hospital, Peschiera del Garda, Italy; ^4^Department of Neurology, Hochzirl Hospital, Zirl, Austria; ^5^Research Unit of Neurorehabilitation, South Tyrol, Bolzano, Italy; ^6^Department of Rehabilitation, Brunico Hospital, Brunico, Italy; ^7^Neurorehabilitation Unit, Azienda Ospedaliera Universitaria Integrata, Verona, Italy

## Abstract

Association between the site of brain injury and poststroke spasticity is poorly understood. The present study investigated whether lesion analysis could document brain regions associated with the development of severe upper limb poststroke spasticity. A retrospective analysis was conducted on 39 chronic stroke patients. Spasticity was assessed at the affected upper limb with the modified Ashworth scale (shoulder, elbow, wrist, and fingers). Brain lesions were traced from magnetic resonance imaging performed within the first 7 days after stroke and region of interest images were generated. The association between severe upper limb spasticity (modified Ashworth scale ≥2) and lesion location was determined with the voxel-based lesion-symptom mapping method implemented in MRIcro software. Colored maps representing the *z* statistics were generated and overlaid onto the automated anatomical labeling and the Johns Hopkins University white matter templates provided with MRIcron. Thalamic nuclei were identified with the Talairach Daemon software. Injuries to the insula, the thalamus, the basal ganglia, and white matter tracts (internal capsule, corona radiata, external capsule, and superior longitudinal fasciculus) were significantly associated with severe upper limb poststroke spasticity. Further advances in our understanding of the neural correlates of spasticity may lead to early targeted rehabilitation when key regions are damaged.

## 1. Introduction

Spasticity is characterized by a velocity-dependent increase in muscle tone with exaggerated tendon jerks, resulting from hyperexcitability of the stretch reflex [1]. It affects neuromotor function after a lesion in the corticospinal [2] and accompanying parapyramidal (corticoreticulospinal) pathways [2].

Poststroke spasticity (PSS) has a prevalence ranging from 4–27% in the early phase of illness (1–4 weeks after stroke) to 17–43% in the chronic one (>3 months) (no clear difference between the upper and lower limbs, but a frequently higher severity in the former) [2, 3]. Severe PSS [3] carries a heavy burden, as it is associated with pain, decreased quality of life, higher impairment, and disability [4].

Low Barthel Index (BI) score [5], marked paresis [3–6], poststroke pain [6], and sensory deficits [3] were reported to relate to the presence and severity of PSS [2], but the heterogeneous measures of spasticity and the variable findings from previous studies limit their strength as early predictors. Stroke location was consistently reported to play a role in upper limb motor recovery [7], but its association with upper limb PSS is unclear.

Elucidating this point could enhance our understanding of the neural circuitry involved in spasticity and help prioritizing prevention or early treatment in patients at high risk for developing PSS. Thus, the aim of the current investigation was to determine the association between stroke lesion location and severe upper limb PPS using brain voxel-based lesion-symptom mapping (VLSM) procedures [8].

## 2. Materials and Methods

In this retrospective study, we recruited 116 adults, who consecutively were referred to the Neurorehabilitation Unit of the Azienda Ospedaliera Universitaria Integrata of Verona (January 2011–December 2012) for the evaluation of a recent stroke.

Inclusion criteria are as follows: first-ever unilateral ischemic stroke occurred 3–6 months earlier and high resolution 1.5 T anatomical magnetic resonance imaging (MRI) scans were performed within the first 7 days after stroke with T2-weighted fluid-attenuated inversion-recovery (FLAIR) and diffusion-weighted imaging (DWI) sequences available. Exclusion criteria are as follows: previous stroke, other neurological disorders or musculoskeletal conditions that might bias spasticity evaluation, and previous or current treatment with antispastic drugs or therapies (e.g., botulinum toxin, phenol block) that might influence spasticity. Patients were treated according to the current stroke rehabilitation guidelines [9].

All patients gave their written consent for participation in the study. The protocol was carried out according to the declaration of Helsinki and was approved by the Ethics Committee of the Department of Neurological and Movement Sciences of Verona University.

### 2.1. Muscle Tone Assessment

Spasticity was assessed with the modified Ashworth scale (MAS) [3, 10], which is a 6-point scale grading the resistance of a relaxed limb to rapid passive stretch (0: no increase in muscle tone; 1: slight increase in muscle tone manifested by a catch and release or by minimal resistance at the end of the range of motion when the affected part(s) is moved in flexion or extension; 1+: slight increase in muscle tone manifested by a catch, followed by minimal resistance throughout the remainder (less than half) of the range of motion; 2: more marked increase in muscle tone through most of the range of motion; 3: considerable increase in muscle tone, passive movement difficulty; and 4: affected part(s) rigid in flexion or extension). Spasticity evaluation at the affected arm included passive flexion and extension movements of several joints with the patient in a sitting position. We tested arm abductors/adductors, elbow flexors/extensors, wrist flexors/extensors, and finger flexors.

Patients were divided into those with (SP+) and those without (SP–) PSS, which was defined as MAS ≥ 2 for any of the tested movements [3].

### 2.2. Lesion Tracing

Lesions were visually identified as having altered FLAIR and DWI signal intensity compared to corresponding contralateral tissue. DWI sequences were used when MRI was performed within the first 48 h after stroke and FLAIR sequences when MRI was performed between 48 h and 7 days after stroke [11]. Lesion tracing was carried out using the ch2bet anatomical brain template provided with the MRIcro software (http://www.mricro.com/) and region of interest (ROI) images were generated [8]. Furthermore, ROI images of each patient were converted into volume of interest (VOI) images using MRIcron software (http://www.mricro.com/mricron). All lesions were traced by a trained image analyst and confirmed by an experienced clinical neurologist, who was blind to all clinical data, except for the side of hemiparesis.

Since the proportion of right and left hemisphere lesions was not significantly different between the SP+ and SP− groups, and to improve the power of the lesion overlays analysis, ROI images were transformed to the right hemisphere [12]. We felt confident that this procedure would not affect our results because previous studies suggested that lesion laterality does not significantly influence PSS [3, 13, 14].

### 2.3. Lesion and Statistical Analysis

Association between severe upper limb PSS and VOI images was analyzed with the VLSM methods implemented in the nonparametric mapping (NPM) software included into the MRIcron software [8].

For statistical purposes, we considered severe PSS as a binary variable (absent/present) according to a binary images/binary behaviour design [8]. Voxels damaged in less than 2% of patients were ignored. The nonparametric Liebermeister statistical analysis for binary data was used [8]. The level of significance was *P* < 0.05 and corrected for multiple comparisons with the false discovery rate (FDR) threshold (permutation thresholding was reported to be less sensitive than FDR and consequently was not performed) [15].

Colored VLSM maps representing the *z* statistics were generated and overlaid onto the automated anatomical labeling (AAL) and the Johns Hopkins University (JHU) white matter templates provided with MRIcron software [8]. Since MRIcron software does not provide information on thalamic divisions, the Talairach Daemon software (http://www.talairach.org/) was used for the overlaid analysis of thalamic nuclei [16].

Pearson's chi-squared test with Yates' continuity correction was used for frequencies, Mann-Whitney *U* test for continuous variables, and Spearman rho for correlations for the analysis of patients' demographic clinical characteristics (*P* < 0.05).

## 3. Results

**Figure 1:**
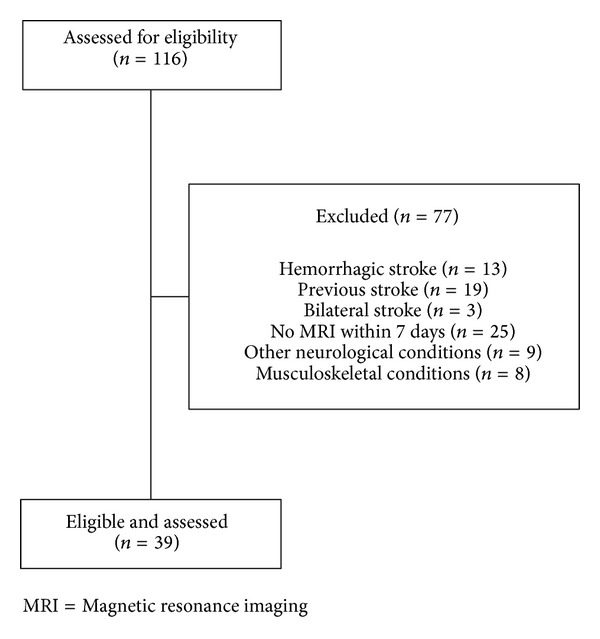
Flow diagram of the study.

**Figure 2:**
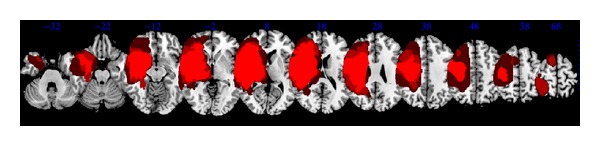
Overlay of lesions for all patients.

Thirty-nine patients (24 males, 15 females; mean age 72.7 years) were included (Figure 1). Among the demographic (age, sex) and clinical characteristics (mean time from stroke: 4.5 months; side of stroke: 21 left, 18 right; and location of stroke: cortical 8, subcortical 12, and mixed 19), only stroke severity was significantly greater in the SP+ group (European Stroke Scale: SP+: 51.6 ± 11.9, SP−: 88.6 ± 8.7, *P* < 0.001; Table 1). Overlay of lesions for all patients is shown in Figure 2.

**Figure 3:**
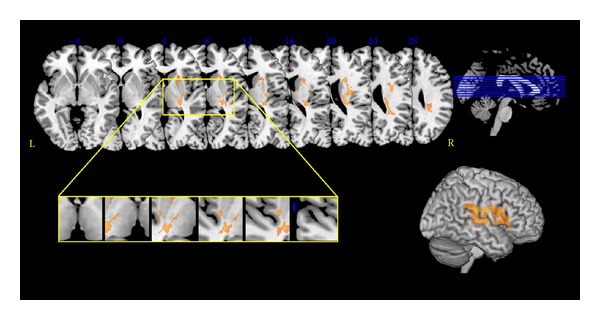
Statistical voxel-based lesion-symptom mapping. The nonparametric Liebermeister statistical analysis was used for the binary variable severe poststroke spasticity. Here all voxels that survived a 1% false discovery rate cut-off threshold are reported. The yellow box highlights the distribution of thalamic significant voxels, which corresponded to the ventral posterior nucleus in the Talairach atlas.

The number of MRI voxels involved by the stroke lesion was significantly larger in the SP+ (63750.2 ± 75764.1, median 33799) than in the SP− group (16298.4 ± 31706.7, median 4124.5, *P* = 0.001; Table 1). The lesion overlay of SP+ patients and the VLSM revealed cerebral areas that were significantly associated with severe PSS (Figure 3, Table 2).

Grey matter areas included the insula, the basal ganglia (caudate, putamen, and pallidum), and the thalamus. White matter tracts included the anterior and the posterior limb, the retrolenticular part of the internal capsule, the anterior, superior, and posterior corona radiate, the external capsule, and the superior longitudinal fasciculus. The thalamic areas significantly correlated with severe PSS corresponded to the ventral posterior lateral nucleus in the Talairach atlas (Figure 3).

## 4. Discussion

The current study explored the anatomy underlying the development of spasticity after stroke and documented that damage to the insula, the thalamus, the basal ganglia, and many white matter tracts (internal capsule, corona radiata, external capsule, and superior longitudinal fasciculus) is associated with severe upper limb PSS.

We used a bottom-up lesion analysis, where patients were grouped according to the presence of severe upper limb PSS rather than lesion location. Our approach reduced the chance of overlooking structures outside arbitrarily predefined ROIs [7] and identified the critical areas with 1 mm resolution. It also overcame inaccuracies and uncertainties of previous anatomical studies, which defined lesion locations very broadly by categorizing them as cortical versus subcortical, or by computerized tomography scan instead of MRI [17].

Damage to corona radiata and internal capsule was previously found to predict motor recovery after stroke, while pure cortical lesions did not [7]. In keeping with previous data, we did not find a correlation between severe PSS and cortical strokes. Shared pathophysiological mechanisms between spasticity and paresis explain why marked paresis [3–6] is a good clinical predictor of severe PSS. Our findings however indicate that severe PSS behaves differently from paresis, in that the latter was found to correlate with the damage of the posterior limb of the internal capsule [7], while we found the former to be associated with lesions to different components of the internal capsule and corona radiata. We may speculate that the involvement of more primitive rubrospinal, reticulospinal, and vestibulospinal motor control systems [7] may have contributed to severe PSS in our patients.

This is the first report of a correlation between stroke in the basal ganglia as well as in the external capsule and severe PSS. The basal ganglia play a central role in motor control. In that, they have bidirectional connections with the primary motor cortex, premotor, and supplementary motor areas through basal ganglia-thalamocortical circuits. Damage to the basal ganglia might contribute to the spastic dystonia component, which is common in patients with severe PSS. At variance, the role of the external capsule is difficult to explain on pathophysiological grounds, in that it contains fibres not belonging to the motor system. An explanation for our finding is that these subcortical structures (basal ganglia, external capsule) turned out to be significantly associated with severe PSS because they share the same vascular supply with the internal capsule through the lenticulostriate branches of the middle cerebral artery. Their role in the pathogenesis of severe PSS should be better explored in future studies.

The ventral posterior lateral nucleus of the thalamus and the insula turned out to be significantly associated with severe PSS. This is the first report of the role of the ventral posterior lateral nucleus and the insula in PSS, but our findings are in keeping with the association between PSS and sensory deficits [3] and poststroke pain [6], both of which are common after stroke involving these areas. They are also in accordance with the view that pain may worsen spasticity [2].

The superior longitudinal fasciculus is the major dorsally located fibre pathway linking parietal and frontal cortices [11]. This fasciculus has never been found to be associated with PSS, but its role in spasticity is suggested by diffusion tensor imaging evidence of its alteration in the spastic ataxia of Charlevoix-Saguenay, a rare autosomal recessive neurodegenerative disorder [18].

Taken together, our data show a positive correlation between severe PSS development and the degree of destruction or disorganization of the central sensorimotor system [2]. This finding is in keeping with the significantly larger number of lesioned voxels in the SP+ group and with previous reports showing that BI may predict PSS [5].

Limitations of our study are the small sample size, the absence of correlation between clinical and MRI data, and the use of a single subjective measure of spasticity. Furthermore, the study design might have resulted in the exclusion of patients with minor and less severe strokes (who usually are not referred for neurorehabilitation consultancy), those with most severe strokes (who die before 3–6 months after stroke), or older ones (in these patients computed tomography scan is usually performed instead of MRI). However, the patients' population included in the present study (intermediate stroke severity) is the one where the prediction of PSS is clinically more difficult and has more rehabilitative implications. Finally, lesions were drawn on DWI for some subjects and FLAIR for others because of the different timing of MRI and the original brain scans were of lower resolution than the high resolution T1 scans used in studies in chronic stroke. However, the study was aimed at exploring whether MRI early after stroke may help to predict PSS and, in this phase, the extent of stroke cannot be reliably measured in T1 scans.

## 5. Conclusions

Our results have clinical implications, in the fact that they may help early identification of those patients who carry a higher risk of developing spasticity and may particularly benefit from preventive and therapeutic strategies. Future prospective larger studies including further clinical measures are needed to strengthen our data and better explore predictors of PSS that may lead to early targeted rehabilitation when key regions are damaged.

## Figures and Tables

**Table 1:** Demographic, clinical, and MRI characteristics of the patients.

Patient	Age	Sex	Time from stroke (mos)	Stroke side	Stroke location	ESS score	Lesion voxels (*n*)
1, SP−	65	M	3	L	Subcortical	83	90067
2, SP−	75	F	4	R	Subcortical	80	898
3, SP−	80	F	5	L	Subcortical	94	1584
4, SP−	75	F	6	L	Subcortical	92	11377
5, SP−	67	F	3	R	Mixed	81	4264
6, SP−	78	M	4	R	Cortical	88	8171
7, SP−	83	M	5	L	Mixed	81	3561
8, SP−	76	F	4	L	Subcortical	97	4829
9, SP−	67	M	6	L	Subcortical	81	3558
10, SP−	59	M	3	R	Mixed	88	2455
11, SP−	63	F	4	R	Mixed	80	44832
12, SP−	83	M	5	R	Cortical	100	4264
13, SP−	68	M	6	L	Mixed	83	6299
14, SP−	76	M	4	R	Subcortical	84	2265
15, SP−	73	M	3	L	Subcortical	96	44575
16, SP−	80	F	4	R	Mixed	90	2544
17, SP−	69	M	6	L	Cortical	70	1626
18, SP−	75	M	6	L	Mixed	96	3985
19, SP−	66	M	5	L	Subcortical	85	4829
20, SP−	64	M	5	L	Cortical	100	8171
21, SP−	77	F	6	R	Mixed	78	3561
22, SP−	85	F	4	R	Cortical	100	130059
23, SP−	56	M	3	R	Mixed	100	3177
24, SP−	60	F	4	L	Mixed	100	210
25, SP+	78	F	3	R	Cortical	53	13645
26, SP+	66	F	6	L	Mixed	50	290752
27, SP+	72	M	4	L	Subcortical	64	110211
28, SP+	75	M	4	R	Cortical	62	73818
29, SP+	69	M	3	L	Mixed	61	60229
30, SP+	67	M	3	L	Mixed	58	33799
31, SP+	73	M	4	R	Mixed	51	3050
32, SP+	74	M	6	R	Cortical	61	3251
33, SP+	78	F	5	L	Mixed	58	145595
34, SP+	79	F	5	L	Mixed	30	89151
35, SP+	66	F	6	L	Subcortical	64	60102
36, SP+	84	M	6	L	Subcortical	53	26852
37, SP+	76	M	3	R	Mixed	34	17465
38, SP+	80	M	4	R	Mixed	30	10276
39, SP+	78	M	5	R	Mixed	45	18057

SP−: patient without severe spasticity; SP+: patient with severe spasticity; M: male; F: female; R: right; L: left; ESS: European Stroke Scale.

**Table 2:** Brain regions associated with severe upper limb spasticity.

Region	*x*	*y*	*z*	LB *z* max	*n* voxels
Insula	33	−7	20	3.784	12
Caudate	19	−13	21	3.827	56
Putamen	28	4	13	3.827	256
Pallidum	22	5	2	3.467	8
Thalamus	22	−17	4	3.467	65
Anterior limb of internal capsule	21	22	0	3.643	10
Posterior limb of internal capsule	26	−25	17	3.827	136
Retrolenticular part of internal capsule	23	−25	4	3.467	174
Anterior corona radiate	21	23	−1	3.643	149
Superior corona radiate	29	−6	19	4.188	226
Posterior corona radiate	26	−25	19	3.827	184
External capsule	28	4	13	3.827	159
Superior longitudinal fasciculus	30	−5	21	4.188	133

For each region, the Montreal Neurological Institute coordinates of the centre of mass are provided along with the maximum Liebermeister (LB) *z *statistic in each cluster and the number (*n*) of clustering voxels that survived the threshold of *P* < 0.05, false discovery rate corrected.
